# Glyoxalase 1 copy number variation in patients with well differentiated gastro-entero-pancreatic neuroendocrine tumours (GEP-NET)

**DOI:** 10.18632/oncotarget.20290

**Published:** 2017-08-16

**Authors:** Mingzhan Xue, Alaa Shafie, Talha Qaiser, Nasir M. Rajpoot, Gregory Kaltsas, Sean James, Kishore Gopalakrishnan, Adrian Fisk, Georgios K. Dimitriadis, Dimitris K. Grammatopoulos, Naila Rabbani, Paul J. Thornalley, Martin O. Weickert

**Affiliations:** ^1^ Division of Translational Medicine, Clinical Sciences Research Laboratories, Warwick Medical School, University of Warwick, University Hospital, Coventry, U.K; ^2^ Faculty of Applied Medical Sciences, Taif University, Taif, Kingdom of Saudi Arabia; ^3^ Department of Computer Sciences, University of Warwick, Coventry, U.K; ^4^ University Hospitals Coventry & Warwickshire NHS Trust, The ARDEN NET Centre, ENETS CoE, Coventry, U.K; ^5^ Warwick Systems Biology Centre, Senate House, University of Warwick, Coventry, U.K; ^6^ Coventry University, Centre for Applied Biological & Exercise Sciences, Coventry, U.K

**Keywords:** Glyoxalase, multi-drug resistance, glycation, copy number variation, gastro-entero-pancreatic neuroendocrine tumour

## Abstract

**Background:**

The glyoxalase-1 gene (GLO1) is a hotspot for copy-number variation (CNV) in human genomes. Increased GLO1 copy-number is associated with multidrug resistance in tumour chemotherapy, but prevalence of GLO1 CNV in gastro-entero-pancreatic neuroendocrine tumours (GEP-NET) is unknown.

**Methods:**

GLO1 copy-number variation was measured in 39 patients with GEP-NET (midgut NET, n = 25; pancreatic NET, n = 14) after curative or debulking surgical treatment. Primary tumour tissue, surrounding healthy tissue and, where applicable, additional metastatic tumour tissue were analysed, using real time qPCR. Progression and survival following surgical treatment were monitored over 4.2 ± 0.5 years.

**Results:**

In the pooled GEP-NET cohort, GLO1 copy-number in healthy tissue was 2.0 in all samples but significantly increased in primary tumour tissue in 43% of patients with pancreatic NET and in 72% of patients with midgut NET, mainly driven by significantly higher GLO1 copy-number in midgut NET. In tissue from additional metastases resection (18 midgut NET and one pancreatic NET), GLO1 copy number was also increased, compared with healthy tissue; but was not significantly different compared with primary tumour tissue. During mean 3 - 5 years follow-up, 8 patients died and 16 patients showed radiological progression. In midgut NET, a high GLO1 copy-number was associated with earlier progression. In NETs with increased GLO1 copy number, there was increased Glo1 protein expression compared to non-malignant tissue.

**Conclusions:**

GLO1 copy-number was increased in a large percentage of patients with GEP-NET and correlated positively with increased Glo1 protein in tumour tissue. Analysis of GLO1 copy-number variation particularly in patients with midgut NET could be a novel prognostic marker for tumour progression.

## INTRODUCTION

**Figure 1:**
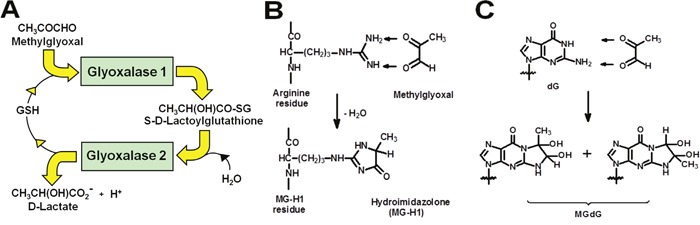
The glyoxalase metabolic pathway and prevention of glycation of protein and DNA by methylglyoxal **(A)** Metabolism of MG by the glyoxalase system. **(B)** Protein modification by methylglyoxal with formation of arginine-derived hydroimidazolone, MG-H1. **(C)** DNA modification by methylglyoxal with formation of deoxyguanosine-derived imidazopurinone MGdG. The adduct residue is shown with guanyl base only. This figure is reproduced with permission from [55]. The major MG glycation adducts of protein and DNA are shown, accounting for *ca*. 90% of total adducts formed. Other minor adducts were described elsewhere [41, 56]

Glyoxalase 1 (Glo1) is part of the cytosolic glyoxalase system present in all human cells. Glo1 catalyses the glutathione-dependent metabolism of the reactive metabolite methylglyoxal (MG) – Figure 1A. MG is formed mainly by the low-level spontaneous degradation of triosephosphate intermediates of anaerobic glycolysis [1]. It is a potent glycating agent of protein and DNA, forming mainly the arginine-derived hydroimidazolone adduct of arginine residues, MG-H1, in proteins; and mainly a mixture of isomeric imidazopurinones, MGdG, of DNA – Figures 1B and 1C [2, 3]. MG-derived adducts of protein lead to protein inactivation and dysfunction and adducts of DNA are associated with DNA strand breaks and mutagenesis [4]. Glo1 suppresses the concentration of MG to low levels and thereby protein and DNA adducts are also suppressed to low, tolerable levels in protein and DNA – *ca*. 1 – 5% of protein and 1 in 10^5^ nucleotides in DNA [3, 5, 6]. In an animal model of hepatocellular carcinogenesis, GLO1 was found to be a tumour suppressor gene, suggesting that MGdG-linked mutations on some occasions lead to cell transformation and malignancy [7]. In established tumours increased Glo1 expression is a mediator of multidrug resistance (MDR) [8], indicating that MG-mediated cytotoxicity may contribute to the mechanism of action of antitumour agents – possibly by induction of apoptosis and anoikis (cell detachment stimulated apoptosis) [9–11]. Cell permeable inhibitors of Glo1 are potential anti-tumour agents and counter Glo1-overexpression mediated MDR [12] but none have yet been developed for clinical use – reviewed in [11, 13].

The human GLO1 gene is located in chromosome 6 at locus 6p21.2 [14]. It consists of 12 kb with five introns separating six exons [15]. It is a hotspot for copy number variation (CNV) in non-malignancy. In constructing a first-generation CNV map of the human genome, Redon *et al*. found a total of 1,447 CNV regions covering 12% of the genome. GLO1 was the only gene found in a copied region of *ca*. 122 kb at *ca*. 2% prevalence [16]. GLO1 CNV was confirmed in a further human population study [17] and also found in other primates and mice [18–20]. GLO1 CNV is functional – increasing Glo1 expression by 3 – 4 fold in all tissues and cells tested [18]. Importantly, GLO1 undergoes amplification in human tumours [21]. In a survey of 520 human tumours, increased GLO1 copy-number was found at a mean prevalence of 8%. The highest prevalence was in breast cancer (19%), small cell lung cancer (16%) and non-small cell lung cancer (11%) [21]. The prevalence of GLO1 copy-number increase in patients with gastro-entero-pancreatic neuroendocrine tumours (GEP-NET) has not been investigated to date.

GEP-NETs develop from neuroendocrine cells of the gastrointestinal tract (GI) mucosa and the pancreatic islet cells. The prevalence of GEP-NETs is thought to be 35 per 100,000 of the population [22], with pancreatic NETs (pNETs) representing approximately one third of cases. The mainstay of clinical treatment is surgical resection of the primary tumour and when possible of metastatic disease [23]. However, as a considerable number of patients with GEP-NETs present with metastatic disease not amenable to surgical resection, further medical treatment is required. Somatostatin analogues represent the first line of treatment for both functioning symptoms and systemic tumour control in patients with well differentiated GEP-NET [24, 25]. Additional therapeutic options include peptide receptor radionuclide therapy and, mainly in patients with pancreatic NET, chemotherapy with alkylating agents such as Streptozotocin (STZ), and treatment with molecular targeted agents such as mammalian target of rapamycin (mTOR) inhibitors and tyrosine kinase inhibitors [13, 26–28]. However, response rates widely vary, with generally better response in patients with pancreatic as compared with midgut NET; and lack of objective response to chemotherapy in up to 60-70% of patients even with pNET [16, 21, 27, 29]. Various prospective predictive factors of tumour response have been proposed for other tumours, but information in patients with GEP-NET is limited and based on studies with relatively low numbers [30], also related to the low prevalence and heterogeneous nature of NET [31]. Identified factors include the proliferation index Ki-67%, Akt, PTEN and thymidylate synthase for streptozotocin-based chemotherapy [30, 32]; and pAKT, PTEN, KRAS, FGFR4 mutations for treatment with mTOR inhibitors [33]. Here, we have analysed GLO1 CNV in a cohort of well characterised patients with GEP-NET who had received curative or debulking surgical treatment. Information about progression and survival was available for a mean 4.2 ± 0.5 years period.

## RESULTS

### Patient characteristics

Characteristics of included patients with suitable histological samples following surgical treatment are shown in Table 1. Data are presented separately for patients with pancreatic NET (n = 14) and midgut NET (n = 25).

**Table 1:** Clinical characteristics of patients with GEP-NET

Characteristic	Pancreatic NET	Midgut NET
n	14	25
Age (mean years; range)	62.8 (39 – 75)	66.2 (34 – 89)
Sex (females/males)	8/6	13/12
BMI (kg/m^2^)	25.4 (19.5 – 34.2)	27.1 (18.6 – 45.2)
Tumour morphology		
Well differentiated	14 (100%)	25 (100%)
Poorly differentiated	0 (0%)	0 (0%)
Tumour grade		
Grade 1	8 (57%)	21 (84%)
Grade 2	5 (36%)	4 (16%)
Grade 3	1 (7%)	
Functioning status		
Functioning (n/%)	1 (7%)	17 (68%)
Non-functioning (n/%)	13 (93%)	8 (32%)
Biomarkers		
Serum chromogranin A (pmol/L)	83.2 ± 31.2	296 ± 103
24-h urine 5-HIAA (μmol/collection)	n/a	142 ± 59
Tumour stating		
Tumour extent		
T1	1 (7.1%)	0 (0%)
T2	3 (21.4%)	5 (20%)
T3	7 (50.0%)	11 (44%)
T4	3 (21.4%)	9 (36%)
Lymph node involvement		
N0	8 (57.1%)	5 (20%)
N1	6 (42.9%)	20 (80%)
Systemic disease		
M0	9 (64.3%)	9 (36%)
M1	5 (35.7%)	16 (64%)
Survival		
Progression free survival (days)	797 ± 217	1456 ± 246
Overall survival (days)	1341 ± 292	1626 ± 264

### GLO1 copy number in neuroendocrine tumour tissue of patients with GEP-NET

**Figure 2:**
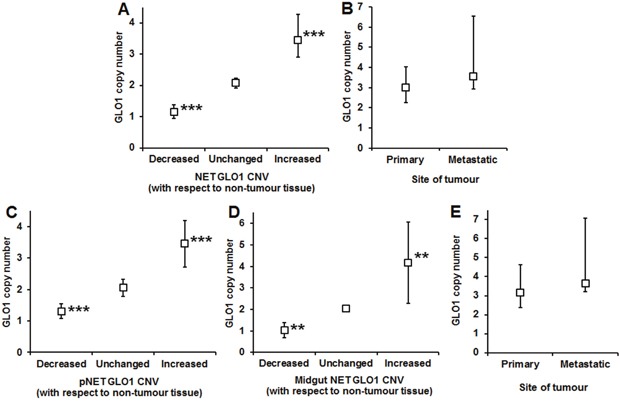
Change in GLO1 copy number in gastro-entero-pancreatic neuroendocrine tumours (GEP-NETs) **(A)** Change of GLO1 copy number in GEP-NETs. Data are median (lower – upper quartile); n = 8 (decreased), n = 6 (unchanged) and n = 25 (increased). **(B)** GLO1 copy number in GEP-NETs – comparison of primary and metastatic tumours. Data are median (lower – upper quartile); n = 19. **(C)** Change of GLO1 copy number in pNETs. Data are mean ± SD; n = 4 (decreased), n = 4 (unchanged) and n = 6 (increased). **(D)** Change of GLO1 copy number in midgut NETs. Data are mean ± SD; n = 3 (decreased), n = 4 (unchanged) and n = 18 (increased). **(E)** GLO1 copy number in midgut NETs – comparison of primary and metastatic tumours. Data are median (lower – upper quartile); n = 18.

Previous estimation of GLO1 copy number in controls had shown the precision of GLO1 determination was (mean ± SD): 2.00 ± 0.13 (n = 21). We therefore assumed copy number >0.39 (3 x SD, covering 99.7% of the probability) was significantly different from healthy control. In pooled analyses (pancreatic and midgut NET combined), GLO1 copy number was increased in GEP-NET tissue, compared with surrounding healthy tissue. GLO1 copy number: mean 3.09, 95% CI 2.52 – 3.66. Number of GEP-NET cases with change in GLO1 copy number, with respect to non-malignant tissue were: decreased GLO1 copy number, n = 8; unchanged GLO1 copy number, n = 6; and increased GLO1 copy number, (n = 25); P<0.001, Kruskal-Wallis. Median [lower – upper quartile] GLO1 copy number in these groups was: unchanged, 2.09 [1.91 – 2.23]; decreased, 1.15 [0.95 – 1.38], P<0.001; and increased 3.46 [2.90 – 4.28], P<0.001 (Figure 2A). Therefore, 64% of GEP-NET had increased GLO1 copy number. For tumours with metastases, GLO1 copy number was not changed between primary and metastatic tumour: primary tumour, 3.00 [2.25 – 4.04]; metastatic tumour 3.55 [2.94 – 6.53], n = 19; Wilcoxon Signed Rank test (Figure 2B).

For pancreatic NET, there were 4 tumours with unchanged GLO1 copy number, 4 tumours had decreased GLO1 copy number and 6 had increased GLO1 copy number (P<0.001). In these groups, GLO1 copy number was: unchanged 2.05 ± 0.28, decreased 1.31 ± 0.23 (P<0.01), and increased 3.45 ± 0.73 (P<0.01); t-test. Therefore, 43% of pancreatic NET had increased GLO1 copy number (Figure 2C). Metastatic tissue was available only from one patient with pancreatic NET, with a low GLO1 copy number of 0.82. Two patients with pancreatic NET were excluded from survival and progression analyses due to early death after surgical intervention; one patient had a high GLO1 copy number in primary tissue (4.13) and one had a low GLO1 copy number (0.91).

For midgut NET, there were 4 tumours with unchanged GLO1 copy number, 3 tumours had decreased GLO1 copy number and 18 with increased GLO1 copy number (P<0.01). In these groups, GLO1 copy number was: unchanged 2.05 ± 0.16, decreased 1.03 ± 0.35 (P<0.01), and increased 4.18 ± 1.89 (P<0.01); t-test. Therefore, 72% of midgut NET had increased GLO1 copy number (Figure 2D). For tumours with metastases, GLO1 copy number was not changed between primary and metastatic tumours. GLO1 copy number was: primary tumour, 3.16 [2.37 – 4.61], metastatic tumour 3.63 [3.22 – 7.06], n = 18; Wilcoxon Signed Rank test, P>0.05. One patient with midgut NET had low GLO1 copy number of 1.24 in metastatic tissue (Figure 2E).

### Immunohistochemistry of glyoxalase 1 protein

We could access further archived sample tissue for immunohistochemistry (IHC) analysis of Glo1 protein in tumour and non-tumour tissue in 29 cases; 7 pNET and 22 midgut NET. Tumour GLO1 copy number was increased, with respect to non-tumour tissue, in 19 of these cases. Glo1 Digital-IHC Score was associated positively with Glo1 IHC staining intensity Pathology Score. For Pathology scores 1 and 2 combined versus 3, Glo1 Digital-IHC Score (arbitrary units) were 1.61 ± 0.33 (n = 14) versus 2.08 ± 0.55 (n = 15), respectively; P<0.01, *t-test*. Overall, there was no correlation of GLO1 copy with Glo1 Digital-IHC intensity in these NET cases. However, for cases showing increased Glo1 Digital-IHC Score in tumour versus non-tumour tissue, there was a positive correlation of change in Glo1 Digital-IHC Score with Glo1 copy number: r = 0.62, P = 0.025; *Pearson* (n = 13). This was also found when cases were selected for increased Pathology Score: r = 0.63, P = 0.016; *Pearson* (n = 14).

### Progression free and overall survival

**Figure 3:**
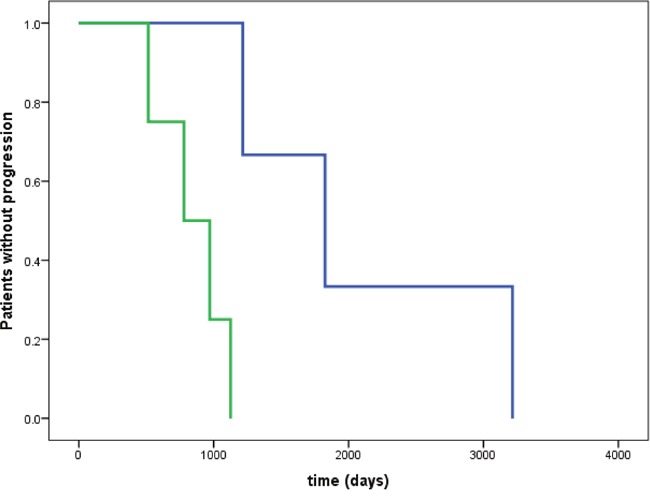
Time without progression in patients with midgut NET Time without progression was significantly longer in patients with normal or low (< 2.4) versus increased (≥ 2.4) GLO1 copy number [log Rank (Mantel-Cox), Chi square 5.629, p = 0.018]. No significant difference in time without progression was observed in patients with pancreatic NET [log Rank (Mantel-Cox), Chi square 0.582, p = 0.46]. Blue line: GLO1 copy number < 2.4; green line: GLO1 copy number ≥ 2.4.

The mean progression free survival was shorter in patients with pancreatic NET, compared with patients with midgut NET (2.2 ± 0.6 versus 4.0 ± 0.7 years; P = 0.018); whereas overall survival was not significantly different between groups (3.7 ± 0.8 versus 4.6 ± 0.7 years; P = 0.51). In patients with midgut NET, time without progression was longer in patients with normal or low (< 2.4) versus increased (≥ 2.4) GLO1 copy-number repeats [log Rank (Mantel-Cox), Chi square 5.629, P = 0.018] (Figure 3). In contrast, in patients with pancreatic NET, time without progression was not significantly different in patients with normal or low (< 2.4) versus increased (≥ 2.4) GLO1 copy-number repeats [log Rank (Mantel-Cox), Chi square 0.582, p = 0.46].

At the time of analysis, 6 (15%) of the here investigated 39 patients with suitable histological samples and complete data had died of disease (Figure 3); of those, 2/25 patients (8%) died related to a midgut NET and 4/14 patients (29%) died related to a pancreatic NET, during a total observation period of up to11 years following surgical treatment of the NET.

### Correlation analyses

There was no significant correlation of GLO1 copy number with Glo1 mRNA in primary GEP-NET tumours (r = 0.20, p = 0.31). In patients with midgut NET, GLO1 copy number in metastatic tumour tissue strongly and significantly positively correlated with chromogranin A concentrations (r = 0.70; p = 0.016), as measured directly before surgical treatment was performed; but a similar correlation of GLO1 copy number with chromogranin A in primary tumour tissue was absent (r = 0.27; p = 0.35). In contrast, in patients with pancreatic NET there was a very strong negative correlation of preoperative chromogranin A with GLO1 copy number in primary tumour tissue (r = - 0.94, p = 0.005).

In the entire cohort, overall survival correlated with tumour stage T (r = 0.33, P = 0.042), but not with functioning status, staging according to nodal or systemic disease, type of surgery performed, tumour grade, age, BMI or sex (all p > 0.11). There was a strong negative correlation of serum Chromogranin A concentrations with BMI (r = - 0.51; P = 0.022).

### Effect of glyoxalase 1 silencing on the growth of pancreatic neuroendocrine tumour BON1 cells *in vitro*

**Figure 4:**
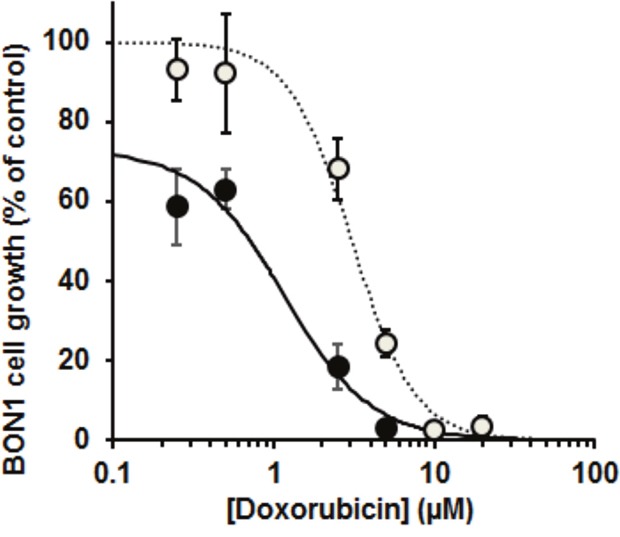
Effect of doxorubicin on the growth of the pancreatic neuroendocrine tumour cell line and effect of silencing of glyoxalase 1 Doxorubicin concentration-response curve for BON1 cell growth *in vitro*. Key: open symbols and dotted line, wild-type Glo1 expression; filled symbols and solid line, BON1 cells with Glo1 silencing. Data are mean ± SEM, n = 3 for Doxorubicin concentrations 0.25, 0.50, 2.5, 5, 10 and 20 μM. Dose-response curve equations given are: wild-type Glo1 expression, BON1 cell growth (% of control) = 100 × 3.06^2.23^/(3.06^2.23^ + [Doxorubicin]^2.23^); and with Glo1 silencing - BON1 cell growth (% of control) = 73 × 1.16^1.61^/(1.16^1.61^ + [Doxorubicin]^1.61^).

To explore if Glo1 expression may be a factor influential in the effectiveness of chemotherapy of NETs, we studied the effect of Doxorubicin, a drug which has been used in combination with others for treatment of NETs – although it is not currently a preferred treatment option [38]. We used the BON1 cell line as an *in vitro* model and knocked down Glo1 expression by siRNA silencing. In control conditions, Doxorubicin inhibited the Growth of BON1 cells: GC_50_ = 3.06 ± 0.13 μM and n = 2.23 ± 0.20. Glo1 silencing potentiated the inhibition of BON1 cell growth by Doxorubicin: with Glo1 silencing, GC_50_ = 1.16 ± 0.13 μM and n = 1.61 ± 0.13. Glo1 silencing alone also decreased BON1 cell growth by 27 ± 2% (n = 3, P<0.001) under the siRNA transfection conditions described (Figure 4).

## DISCUSSION

We present herein the first report of GLO1 copy number variation in GEP-NET and the finding of high prevalence of increased GLO1 copy number – particularly of midgut NETs. Increased GLO1 copy number appeared to be clinically functional through link to increased tumour progression in midgut NET.

Increased GLO1 copy number was previously found in other human tumours where the highest prevalence was in breast cancer, small cell lung cancer and non-small cell lung cancer [21]. Here, we show that in our cohort of patients with well differentiated GEP-NET, the frequency of increased GLO1 copy number was markedly higher than in other tumours investigated to date, with prevalences of 43% in pancreatic NET and 72% in midgut NET, respectively. Comparable GLO1 copy number in metastatic and primary tumour indicated that increased GLO1 copy number, once acquired by the GEP-NET, was maintained. Moreover, increased GLO1 copy number was associated with a significantly shorter time to tumour progression in patients with midgut NET. Increased GLO1 copy number and related expression has previously been considered permissive for tumour growth with high flux of glycolysis and hence flux of formation of MG [3] and also resistance to cancer chemotherapy [8]. We also found decreased GLO1 copy number in 29% of pancreatic NET and 12% midgut NET. This may be due to GLO1 allele deletion or tumour genetic instability; or DNA damage in sample processing, thereby disrupting binding of primers for qPCR.

The observation in our study that Glo1 mRNA expression did not significantly correlate with GLO1 copy number was possibly related to sample size and poor efficiency of mRNA extraction from available FFPE tissue blocks, which is a limitation of this study. Most mRNA and other long RNAs are fragmented during formalin fixed, paraffin-embedded (FFPE) tissues [39]. However, in more than 80% of the respective cases with higher GLO1 copy number, mRNA expression was increased as well. Importantly, in a genome wide copy number variation analysis, GLO1 copy number was among only 3 genes out of 600 investigated where consistently increased gene expression was confirmed in all investigated tissues [18], supporting that GLO1 copy number increases are functional.

Extraction of DNA and protein from FFPE tissue sections is more efficient than for RNA and we therefore sought evidence of association of GLO1 copy number with Glo1 protein in NET tissue by immunohistochemical analysis. Although there was no overall correlation of Glo1 Digital-IHC Score with Glo1 copy number, in patients where Glo1 Digital-IHC Score was higher than in related non-tumour tissue, the increase in Glo1 Digital-IHC Score correlated positively with GLO1 copy number where selection was made by either Glo1 Digital-IHC Score of the stained tissue section or by expert pathologist assessment. The correlation coefficient values, 0.62 – 0.63, suggesting that GLO1 copy number accounts for *ca*. 40% of the variation in Glo1 expression. Therefore, where there is increase in Glo1 expression in NETs, GLO1 copy number increase appears to be an influential factor.

Increased GLO1 copy number is likely a negative survival factor in NETs through its mediation of overexpression of Glo1. The latter is permissive for growth of tumours with high glycolytic rate and related high flux of potentially cytotoxic MG [3]. The clinical treatment of patients in this study was by surgical resection of the primary NET. Residual primary and metastatic tumours with the growth advantage of Glo1 overexpression may impact negatively and markedly so on survival. The mechanism of GLO1 copy number increase in NETs is unknown. Our recent studies suggest it may be driven by hypoxia-activated histone demethylase *KDM4A/*JMJD2A. Increased histone demethylation is hypothesised to create more open chromatin which promotes inappropriate recruitment of mini-chromosome maintenance (MCM) proteins and DNA polymerases and thereby facilitate re-replication of genomic DNA for copy number gain – as recently described [11]. KDM4A is highly expressed in many tumours where it is also involved in metabolic reprogramming for increased tumour anaerobic glycolysis [40].

It might be expected that increased Glo1 expression in NETs would be associated with decreased levels of protein and DNA glycation adducts. This applies, however, if there is no proportionate or disproportionate increase in flux of MG in NETs – as indicated by metabolic modelling of the glyoxalase pathway [27]. Previous experimental studies suggest that tumour cell lines with high GLO1 copy number and Glo1 expression have high glycolytic rate, high flux of MG formation and relatively high content of DNA glycation adducts [3, 21]. This likely reflects an imperfect adaptation of tumour cells with high glycolytic activity to suppress increased MG concentration and potential cytotoxicity. MG adducts of protein and DNA cannot be reliably assessed in FFPE tissue samples as the preservation and analyte extraction procedures compromise the assays [3, 41]. MG protein and DNA adducts may be assessed in NETs in future studies when suitable frozen tumour samples have been accumulated.

To assess the likely functionally of Glo1 expression in chemotherapy of NETs, we studied the effect of doxorubicin, a drug that has been used in combination with others for the treatment of NETs [42], on the growth of pancreatic NET cell line, BON1. Silencing of Glo1 potentiated the growth inhibitory effect of doxorubicin on BON1 cells and, moreover, also decreased BON1 cell growth in the absence of drug treatment. This suggests that Glo1 expression may be a factor linked to resistance of NETs to doxorubicin and likely other anticancer drugs. GLO1 may also suppress cytotoxicity of STZ and cause MDR in chemotherapy of pancreatic NET. Cell permeable Tat-Glo1 protein prevented STZ-induced toxicity to pancreatic beta-cells [43]. Glo1 overexpression was linked to MDR in cancer chemotherapy of other tumour types [8, 12]. Where Glo1 overexpression was found, resistance to cytotoxicity could be overcome by siRNA silencing of Glo1 or inhibition by cell permeable Glo1 substrate analogue inhibitor [12, 21, 44, 45]. Further studies of anticancer drugs with low sensitivity to Glo1-mediated MDR for chemotherapy of NETs may now be considered. The mechanism of Glo1-mediated MDR is likely the suppression of MG-induced apoptosis contributing to the mechanism of action of anticancer drugs – as recently reviewed [11].

A potential role of GLO1 as a predictive factor for both tumour progression and response to treatment could be of particular interest, considering that GLO1 is the most frequently amplified gene in numerous human cancer cell lines [21], with additional GLO1 copies typically being functional [21, 27, 29].

We also found that in patients with midgut NET, a low GLO1 copy number was associated with increased time to tumour progression, thereby possibly representing an independent prognostic factor. A similar effect in patients with pancreatic NET was absent. These results further support the view that pancreatic NET have distinct characteristics as compared with midgut NET, with known differences in both treatment response and overall survival between these types of neuroendocrine malignancies [42, 46–48].

Incidence of NET has 4 – 5 fold increased in Westernized countries from 1973 – 2007, with incidence influenced by age, gender, ethnicity and geographic location [49]. A family history of cancer was a significant risk factor for all NETs. In a USA-based study, a long-term history of diabetes mellitus was a risk factor for midgut NETs (adjusted odds ratio [AOR] = 5.6), particularly in women (AOR = 8.4). Diabetes increased this risk 6-fold in women to AOR = 52.2 [50]. There has recently been intense interest in the increased cancer risk in patients with diabetes. The increase in relative risks associated with diabetes are greatest (≥ 2-fold) for cancers of the liver, pancreas and endometrium [51]. Midgut NET in women appear to also be in this grouping. In the pre-malignant state, Glo1 is a tumour suppressor protein [7], which is likely mediated through suppression of MG modifications of DNA and associated mutagenesis. Diabetes is associated with tissue-specific down regulation of Glo1 and increased MG [52], which may increase risk of NET tumourigenesis. Hyperinsulinemia in type 2 diabetes is also associated with an increase of both estrogen and testosterone in women but not in men – which stimulates cell proliferation and decreases apoptosis [53]. Once malignant transformation is established in NETs, the increased Glo1 activity of NETs may provide a growth advantage that underlies the association of increased GLO1 copy number with poor survival.

Further investigations of the clinical impact of GLO1 copy number in patients with GEP-NET will require multicentre studies with relevant numbers of patients who ideally had surgical on repeated occasions; and patients with surgical samples from tumour resection and subsequent treatment with STZ-based chemotherapy, mTOR inhibitors or tyrosine kinase inhibitors in case of a future relapse or progression. Neoadjuvant chemotherapy for patients with advanced pancreatic NET aiming at tumour downsizing before surgical resection has been recently proposed as well [54] and may provide further opportunities for future research. However, even if a relevant number of cases could be achievable in a large international multi-centre setting, results of GLO1 CNV may be influenced by not rarely observed changes in tumour biology of GEP-NET in the chronic setting, and by influences of the treatment with cytotoxic agents per se, i.e. by increasing cellular MG concentrations and related cytotoxicity as part of their mechanism of action [12, 21, 26].

In further studies it will be important to confirm and corroborate the findings of increased GLO1 copy number in patients with GEP-NET with a related paralogue ratio test and also, in selected samples, examine whether the genetic domain increased in copy number is similar to that found in other tumours, using high intensity genome-wide DNA microarray analyses [21, 29]. With increased patient numbers and long term (> 10 years) follow-up, the association of increased GLO1 copy number and expression with rapid progression may be evaluated in a case-control study. The current study suggests that overexpression of Glo1 through increased GLO1 gene copy number in patients with GEP-NET may facilitate rapid progression and resistance to therapy – assuming a role as it does in other tumour types of mediator of MDR.

In conclusion, increased GLO1 copy number was a very common molecular pathological event in patients with GEP-NET. Measuring GLO1 copy number may provide a novel predictive marker to assess the response to treatment in systemic disease.

## MATERIALS AND METHODS

Samples for analyses were identified from the GEP-NET database in the ARDEN NET Centre, University Hospitals Coventry and Warwickshire (UHCW) NHS Trust, European Neuroendocrine Tumour Society (ENETS) Centre of Excellence. Ethics approval had been obtained from the ARDEN Tissue Bank, Application for Biomaterials (ATB15-004; July 2015; UHCW ethics approval 12/SC/0526). Formalin fixed paraffin embedded (FFPE) tissue blocks from surgical resection of primary tumours and surrounding healthy tissue were available from procedures performed between 2005 and 2015. Only samples with both available tumour tissue and healthy surrounding tissue were selected for analyses. In mixed sections, separate cuts from FFPE tissue blocks were provided for the purpose of the analyses. The respective area of tumour and healthy tissue was determined by a Consultant Histopathologist with extensive experience in GEP-NET and clearly labelled for further analysis. A total of 290 sections and 348 scrolls was received. Two patients with pancreatic NET who died early (< 10 days) after Whipple's procedure were excluded, given the impact on progression and survival analyses. All patients had well differentiated GEP-NET grade 1 or grade 2 GEP-NET, with the exception of one patient with pancreatic NET who had a grade 3 (Ki-67 proliferation index 25%) but also well differentiated tumour. The baseline characteristics of the included patients (pancreatic NET; n = 14; midgut NET, n= 25) are given in Table 1.

### DNA and RNA extraction from tissue sections

*DNA extraction from FFPE sections* For DNA extraction, 2 pieces of 7 ~ 10 μm section scrolls were cut in each block. For tissue sections received in binding on slides, the healthy tissue and tumour tissue was manually scraped respectively in a 1.5 ml Eppendorf tube containing 500μl of 100% ethanol. After centrifugation at 17,000g for 10 min at room temperature, the supernatant was discarded and open tubes were left in 37°C for ethanol to evaporate to dryness. Samples were then incubated twice at 65°C for 15 min with 1ml of xylene to dissolve and remove paraffin and two washes with 100% ethanol to remove residual xylene and left to dry. DNA was extracted with Qiagen DNeasy^®^ Blood & Tissue Kit. The quantity and quality of DNA extraction samples were evaluated with NanoDrop 1000 and stored the DNA at –20°C until further use.

RNA extraction from FFPE sections For each FFPE block, 3 pieces of 7~10 μm section were cut with a microtome and stored in the DNase and RNase free tubes at 4°C until extraction of total RNA. Total RNA was extracted with PureLink FFPE RNA Isolation Kit (Invitrogen) and performed according to the manufacturer's instructions. Briefly, sectioned FFPE tissue deparaffinised with melting buffer and incubated at 72°C for 10 min to melt paraffin. Sections were then digested with proteinase K at 60°C for 4 h and centrifuged (17,000 g, 10 min, room temperature) to sediment no-digested tissue and separate paraffin. The supernatant, tissue lysate, was transferred to a new tube and 400 μl binding buffer and 800 μl 100% ethanol added. The tissue lysate was further processed by selective binding of RNA to a silica-based membrane in a microspin column. Total RNA isolation and purification were performed with thorough washing with buffer and RNA eluted in RNase-free water. The RNA quality and quantity were determined with NanoDrop 1000 and stored the RNA at –80°C until further use.

Both DNA and RNA extraction yielded good quantity and quality of nucleic acid. For pNET, RNA and genome DNA in 17 cases out of 18 cases were successfully extracted and in midgut NET, all extractions from the FFPE samples were successful.

### mRNA analysis with quantitative real-time polymerase chain reaction (qRT-PCR)

Reverse transcriptase reaction was performed in 20 μl total volume with 500 ng total RNA with High-Capacity cDNA Reverse Transcription Kit (Applied Biosystems^™^) and run using an Eppendorf Mastercycler gradient. The reaction was incubated at 25°C for 10 min, then 37°C for 2 h, and then 85°C for 5 min. After 3-fold dilution, reverse transcription product cDNA (2 μl) was used for qRT-PCR to detect target gene expression level with TaqMan technique on ABI 7500 real time PCR system in 20 μl of reaction volume. The initial reaction was at 95 °C for 10 min, followed by 40 cycles at 95°C for 15 s and 60°C for 1 min. The primers and probe for Glo1 and ACTB were pre-designed primers probe mixture from Life Technology Ltd (Paisley, Scotland). Gene expression level was evaluated using 2^(-ddCt)^ with ACTB as a reference gene for normalization for relative expression level. Analyses were performed in triplicate. Values are presented as the relative expression of the target gene in tumor tissue compared to healthy tissue.

### Real-time quantitative PCR for GLO1 copy number estimation

Real-time Quantitative PCR was performed using TaqMan^®^ Copy number assays protocol (Applied Biosystems). The PCR reaction contained: genomic DNA (20 ng), 2x concentrated TaqMan master mix (10 μl), 20x primer-probe working mixture for target gene GLO1 and reference gene RNase P (1 μl) in 20 μl total reaction volume. The PCR reaction was performed with an ABI 7500 real time PCR system. The cycle conditions were: initial cycle, 95°C for 2 min, with following 40 cycles at 95°C for 15 s and 60°C for one min. PCR Reactions were performed in quadruplicate for each sample. The copy number for each sample was calculated with 2^(-ddCt)^ method and the GLO1 copy number in cancer tissue was referenced to healthy tissue assuming GLO1 copy number 2.00. From analysis of GLO1 copy number in the healthy human population, qPCR analysis gave GLO1 copy number 2.00 ± 0.13 (n = 21). GLO1 copy number outside of the interval mean ± SD, 1.61 – 2.39, was considered to be significantly different from the normal control 2.00 copies.

### Tissue immunohistochemistry of glyoxalase 1 protein

Tissue sections (5 μm) of tumour and healthy tissue were cut from formalin-fixed paraffin-embedded (FFPE) tumour and healthy tissue block with a microtome. Sections were immersed in xylene (3 × 5 min), isopropanol (2 × 2 min), 70% (v/v) isopropanol/water (1 × 2 min), and finally rinsed for 2 mins in water. Antigen recovery in the sections was performed with 2100 Antigen Retriever^™^ antigen-retrieval buffer (Aptum Biologics Ltd, Southampton, UK) in 10 mM sodium citrate, 0.05% Tween-20, pH 6.0, according to the manufacturer's instructions. After antigen-retrieval procedure, the slides were rinsed with distilled water (2 × 5 min). For Glo1 immunostaining, VECTASTAIN *Elite* ABC (Rabbit IgG) Kit (Vector Laboratories, Peterborough, U.K.) was used and following the manufacture's user manual. Briefly, sections pre-treated with BLOXALL Endogenous Peroxidase and Alkaline Phosphatase Blocking Solution to block endogenous peroxidase activity for 10 min at room temperature. After washing with tris-buffered saline (250 mM Tris, 27 mM KCl, 1.37 M NaCl, pH 7.4, and 1% Triton X-100; TBST), serum blocking solution was added to sections and incubated at room temperature for 20 min. Excess solution was removed by blotting with filter paper and the sections were incubated with 1:3000 diluted rabbit anti-human GLO1 antibody [34] at 4°C overnight. Slides were then washed with TBST, immersed in biotinylated-anti-human IgG and incubated at room temperature for 30 min. The slides were then washed in PBS, immersed in peroxidase substrate solution, 3,3'-diaminobenzidine (DAB; DAB substrate kit, Abcam, Cambridge) at room temperature 7 min, rinsed with water and then with phosphate buffered Tween (10 mM sodium phosphate, 0.15M NaCl, 0.05% Tween^™^ 20, pH 7.5; PBS-Tween). Slides were counter-stained with Meyer's haematoxylin (Sigma) for 60 s, rinsed in water and then PBS-Tween. The slides were passed through water, 90% EtOH, 100% EtOH and xylene (2 min each) and mounted in DPX solution (Sigma).

The stained slides were assessed by an experienced pathologist (G.K.) and scored for weak, moderate and intense Glo1 immunostaining; scores of 1, 2 and 3, respectively. We also digitised the histology slides using the Mirax Midi^™^ slide scanner (Carl Zeiss MicroImaging GmbH, Jena, Germany). Tissue regions on the digital slides were identified using an entropy based segmentation approach and visual fields containing DAB reactivity above a threshold were selected automatically. This was done to ensure that the automated scoring was restricted to tissue segments containing DAB reactivity only. For antigen quantification, we first digitally reconstructed separate channels for the Haematoxylin and DAB stains in the Optical Density space [35]. Automated quantification of antigen intensity then proceeded as follows [36]: a. Stain intensity was estimated from the DAB channel; and b. Statistics from all selected visual fields in the slide were combined to represent an overall measure (score) for Glo1 protein – referred to as “Digital-IHC Score” below.

### Culture of BON1 pancreatic neuroendocrine tumour cell line, sensitivity to doxorubicin and Glo1 silencing

The BON1 cell line was kindly supplied by Professor Courtney Townsend Jr. (University of Texas Medical Branch, Galveston, TX, USA). BON1 cells were derived from a metastatic human carcinoid tumour of the pancreas and are used as a model of pNET cells. BON1 cells were cultured in Dulbecco's Modified Eagle Medium/Nutrient Mixture F-12 (DMEM/F12) medium containing 2.5 mM L-glutamine, 1:1 (v/v; Thermo Fisher Scientific), supplemented with 5% fetal bovine serum (Sigma) and 1% penicillin/streptomycin (Sigma, Poole, Dorset, U.K.) and incubated in 5% CO_2_ humidified atmosphere at 37°C.

BON1 cells (4 × 10^4^) were seeded in 48-well plates. After culture overnight, cells were transfected with 25 nM Accell Human GLO1 SMART siRNA pool or an Accell non-targeting Control siRNA pool (GE Healthcare Dharmacon Inc, Little Chalfont, Buckinghamshire, U.K.) with Lipofectamine^®^ *RNAiMAX* Transfection Reagent (Thermo Fisher Scientific, Paisley, U.K.) based on the manufacture's protocol. Glo1 silencing was confirmed by assay of Glo1 mRNA which was decreased > 95%. After 72 hours of transfection, the cells were incubated with and without 0.25 – 20 μM doxorubicin. The stock solution of doxorubicin was 10 mM in DMSO such that the maximum exposure to DMSO was 0.2%. Cell growth was assessed by the 3-(4,5-dimethylthiazol-2-yl)-2,5-diphenyltetrazolium bromide (MTT) method [37]. Data were fitted by non-linear regression to the doxorubicin concentration-response equation, A_570,Drug_/A_570,Control_ = 100 x GC_50_^n^/(GC_50_^n^ + [Doxorubicin]^n^), solving for GC_50_ and n where A_570,Drug_/A_570,Control_ is the absorbance at 570 nM in MTT incubated extracts from cell incubations with and without the anticancer drug, GC_50_ is the median growth inhibitor concentration and n is the logistic regression coefficient.

### Statistical analyses

Data are presented as mean ± SD. Continuous variables were tested for parametric distribution using the Kolmogorov-Smirnov test. Significance of mean and median between different groups was evaluated using Student's t test and Mann-Whitney U test, respectively, and significance of difference of medians of variables of tumours at primary and metastatic sites of the same donor were analysed by Wilcoxon's Signed Rank test. Kruskal-Wallis test was used for comparing ordinal or non-normally distributed variables for more than two groups. For nominally scaled variables, Chi-square tests were applied. Correlation analyses were perfomed by Pearson and Spearman methods for parametric and nonparametric data distributions, respectively. Progression free survival was measured from the time of surgical treatment to progression according to RECIST criteria, death or last follow up. Overall survival was measured from the time of surgical treatment to death. Kaplan Meier survival analyses were performed and differences in survival or time to progression were estimated using log rank tests. A p value < 0.05 was considered statistically significant. Data analyses were performed using IBM SPSS Statistics version 22 (Chicago, Illinois, USA).

## Abbreviations

GLO1: glyoxalase-1 gene
GEP-NET: gastro-entero-pancreatic neuroendocrine tumour
MG: methylglyoxal
CNV: copy number variation
MDR: multidrug resistance
STZ: streptozotocin
mTOR: mammalian target of rapamycin
FFPE: formalin fixed, paraffin embedded
IHC: immunohistochemistry
